# Music-based interventions using digital technology for individuals with acquired brain injuries: a scoping review

**DOI:** 10.3389/fpsyg.2025.1532925

**Published:** 2025-02-05

**Authors:** Huiyuan Yu, Xin Lu, Soo Ji Kim

**Affiliations:** ^1^Department of Music Therapy, Graduate School, Ewha Womans University, Seoul, Republic of Korea; ^2^Music Therapy Education, Graduate School of Education, Ewha Womans University, Seoul, Republic of Korea

**Keywords:** acquired brain injury, music, digital technology, scoping review, music therapy

## Abstract

**Background:**

The use of digital technology in music-based interventions for individuals with brain injuries has gained traction, especially post COVID-19, in addressing the need for effective, long-term rehabilitation. This scoping review examines the landscape of digital music-based interventions, focusing on their application in motor and cognitive rehabilitation for patients with brain injuries.

**Methods:**

We conducted a literature search using five academic databases: PubMed, CINAHL, Medline, Google Scholar, and Web of Science. Twenty-six studies met the predefined criteria for inclusion in this review, and these studies were analyzed including digital interventions used.

**Results:**

Twenty-six of 1994 studies were included. Results demonstrated a clear evolution in intervention methodologies, with earlier research focusing on rhythmic and MIDI-based tools, while more recent studies integrated virtual reality, augmented reality, and adaptive feedback mechanisms. The findings showed significant improvements in motor functions, such as upper limb movement and gait, in most of the reviewed studies, as well as some cognitive benefits, particularly when personalized music interventions were applied. However, challenges were noted regarding device complexity, cost, and inconsistent assessment methods across studies.

**Discussion:**

Digital music-based interventions show substantial promise in enhancing motor and cognitive function for individuals with neurological impairments. Nevertheless, barriers such as technological accessibility, the need for patient comfort, and a lack of standardization in assessment remain. Future research should focus on simplifying interfaces, standardizing protocols, and exploring hybrid interventions that combine immersive virtual reality with the adaptability of music therapy to create holistic, patient-centered rehabilitation solutions.

## 1 Introduction

Acquired Brain Injury (ABI), defined as brain damage occurring after birth, is a significant global health concern. ABI is broadly categorized into traumatic brain injury (TBI) and non-traumatic brain injury (Non-TBI), each with distinct etiologies and implications (Burns and Hauser, 2003). TBI results from external mechanical forces, such as motor vehicle accidents, falls, sports-related injuries, or violence, causing direct damage to brain tissue. In contrast, Non-TBI arises from internal processes, including stroke, neoplasms, infections, or anoxia, which similarly lead to brain damage (Goldman et al., 2022). The consequences of ABI are profound, often resulting in significant limitations to individuals’ daily functioning, employment, and physical activities. Addressing these challenges necessitates comprehensive rehabilitation approaches targeting physical, communicative, behavioral, psychosocial, and environmental domains (Turner-Stokes et al., 2015). Over the past two decades, the global prevalence of ABI has risen considerably, particularly among older adults and in high-income countries (Chan et al., 2013; Majdan et al., 2016). These trends highlight the urgent need for specialized, evidence-based interventions tailored to diverse populations to optimize recovery and minimize long-term disability (Winter et al., 2022).

In rehabilitation treatment for patients with brain injuries, including music interventions, various digital technologies are being utilized. The COVID-19 pandemic highlighted the need to provide long-term rehabilitation even when health centers are closed or patients are unable to travel to treatment centers (Aulisio et al., 2020), and this ignited interest in digital technology. Consequently, healthcare providers are increasingly using digital technologies in rehabilitation treatment. A review of studies on rehabilitation using digital technologies indicates the use of virtual reality (VR), mobile apps, web-based interventions, and electronic strength training for functional rehabilitation (Edwards et al., 2022). A meta-analysis focusing on VR-based rehabilitation therapy after stroke presented statistically significant improvements in upper limb function, functional independence, quality of life, spasticity, and dexterity compared to conventional occupational therapy (Khan et al., 2024). However, the body of research lacks well-defined guidelines regarding the optimal characteristics of VR system, such as immersive versus non-immersive environments, and the most effective feedback mechanisms, including real-time movement correction, performance-based visual cues, or haptic feedback (Høeg et al., 2021). Similarly, the expanding field of music-based interventions using digital technologies lacks standardized guidelines and feedback systems that address the needs of both patients and therapists, underscoring the necessity for user-centered approaches to achieve specific therapeutic outcomes (Kim et al., 2020).

The neurophysiological basis of music interventions in brain injury rehabilitation is deeply rooted in the principle of neuroplasticity in which the brain adapts and reorganizes itself through repeated exposure to stimuli and behaviors (Carey et al., 2019; Vik et al., 2018). Music, particularly when integrated with digital technologies, enhances this process by providing multisensory input—auditory, visual, and tactile—that expands attention and induces repetitive actions, which are key elements in neuroplastic adaptation. Studies have demonstrated that multisensory experiences involving music can strengthen neural pathways by combining auditory and motor activities, fostering both motor recovery and cognitive engagement (Gaser and Schlaug, 2003; Schlaug, 2015). Moreover, audio visual and haptic feedback, as exemplified in music therapy using VR, can redirect attention from internal stimuli to external cues, thereby promoting relaxation, engagement, and motor recovery (Gerber et al., 2017; Naef et al., 2022). These findings highlight the critical role of music-based digital interventions in leveraging neuroplasticity to optimize rehabilitation outcomes.

Previous research indicates successful integration of digital technology into music-based interventions, aiding in the creation of customized treatment plans that meet the needs of both music therapists and patients. The use of digital musical instruments, in particular, has revolutionized the ways music is created and experienced, providing a space for creative expression and real-time acoustic feedback, which is especially beneficial for patients with physical limitations or lacking musical skills (Magee and Burland, 2008). Studies on music-based interventions using digital technologies show that digital instruments, by integrating auditory, tactile, and motor sensations, enhance motor function through repeated and precise movements. Additionally, these digital instruments facilitate activities such as ensemble playing and collaborative music creation, which significantly contribute to improving social interaction and fostering teamwork among participants. These social benefits are particularly evident in group-based music therapy settings, where shared musical experiences help build a sense of community and emotional connection among participants (Partesotti et al., 2018). Additionally, one of the advantages of using digital technology is the ability to more accurately record and analyze a patient’s progress during music-based interventions, helping therapists to more precisely assess and understand the current needs of patients (Ward et al., 2019).

However, a systematic analysis of studies utilizing digitalized musical instruments for acquired brain injury patients indicates a lack of comprehensive research on the use of digital technology in this context across different age groups. There remains a shortage of in-depth studies investigating how standardized assessment tools and intervention designs can be effectively tailored to the needs of acquired brain injury patients of various ages. Moreover, there is limited understanding of how different types of feedback mechanisms—such as visual, auditory, or tactile—can enhance musical experiences and ultimately improve rehabilitation outcomes for diverse patient populations (Chuah et al., 2024). Therefore, further exploration is necessary to validate the therapeutic effectiveness of digital technology-based music activities in the rehabilitation of acquired brain injury patients across the lifespan.

## 2 Method

A scoping review serves to systematically map the extant evidence within a designated research area, elucidate fundamental concepts and definitions, and critically discern gaps in the literature (Thomas et al., 2017). We followed the methodological framework proposed by Arksey and O’Malley (2005) and its subsequent updates (Cooper et al., 2021; Levac et al., 2010). The stages were (1) identifying the research questions; (2) establishing inclusion and exclusion criteria; (3) identifying relevant studies that meet study criteria studies; (4) charting the data; and (5) collating, summarizing, and reporting the data. This review was conducted and reported using the Preferred Reporting Items for Systematic Reviews and Meta-Analyses (PRISMA) Extension for Scoping Reviews checklist (Tricco et al., 2018).

### 2.1 Step 1: identifying research questions

A broad and creative discussion was held to identify relevant research questions, and this discussion incorporated the scientific and empirical knowledge of the researchers. Our focus was on music-based interventions using digital technology for individuals with acquired brain injuries. A narrowing of research questions was performed until a consensus was reached. Our research questions were as follows:

1) What types of music-based interventions using digital technology are utilized for individuals with acquired brain injuries, and what are their primary therapeutic goals?
2) What digital tools and methodologies are commonly used in these interventions, and what indicators or evidence are used to evaluate their impact on therapeutic outcomes?

### 2.2 Step 2: establishing inclusion and exclusion criteria

Each study included in this review met the following inclusion criteria: (a) intervention studies – Focus should remain on music-based interventions that incorporate digital technology. Expand this to include detailed description of intervention protocols, even if outcomes have not been reported; (b) participants had a primary diagnosis of traumatic brain injury (TBI) or non-traumatic brain injury (nTBI), (c) original research articles published in peer-reviewed journals or as conference proceedings, (d) study published in English, and (e) studies reporting measurable outcomes related to therapeutic goals. Studies were excluded if they met any of the following criteria: (a) systematic reviews, meta-analyses, book chapters, or gray literature; (b) studies without accessible full-texts in English; (c) studies focusing on instrument or software development without reporting empirical data; (d) reviews or summary reports that discussed therapeutic use of music without detailed descriptions of interventions; and (e) studies that failed to provide sufficient information on the therapeutic aspects of music, such as interventions conducted without a clear therapeutic framework. When duplicate data were identified across multiple publications, the study with the most comprehensive dataset was retained, while others were excluded to avoid redundancy.

### 2.3 Step 3: identifying relevant studies that meet study criteria

To ensure a comprehensive review, the search strategy was developed collaboratively by all authors through multiple discussions to refine keywords and search terms. The strategy focused on three key concepts: music-based interventions, digital technologies, and brain injuries. Relevant keywords included terms following terms: “Music,” “Instrument,” “Playing,” and “Rhythm”; “Digital,” “AI,” “Virtual Reality,” and “Online Therapy”; and “Parkinson,” “Stroke,” and “Acquired Brain Injury (ABI).” Boolean operators (AND/OR) and truncation symbols (*) were used to maximize search sensitivity. Searches were conducted in CINAHL, Medline, PubMed, Web of Science Core Collection, and Google Scholar. Search fields included titles, abstracts, and subject-specific indexing terms (e.g., Medical Subject Headings [MeSH]).

Searches were first conducted in March 2023 and updated in January 2025 to include newly published studies. Search terms and strategies were iteratively refined, and search results were exported to reference management software to remove duplicates. A full list of search strings is provided in Supplementary material S1.

**Figure 1:**
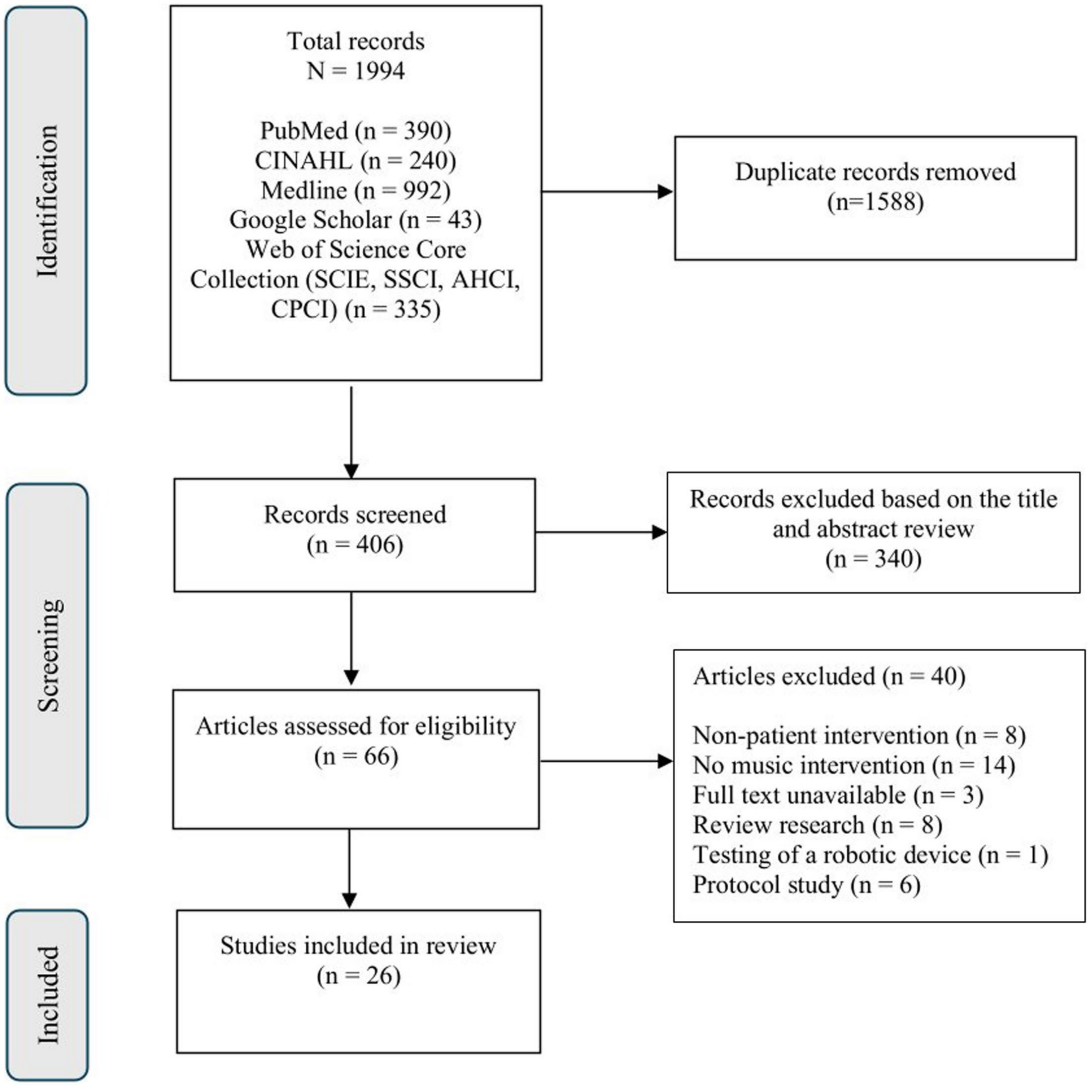
PRISMA flowchart describing the selection process for the included articles.

The study selection process involved two authors (HY and XL) independently performing an initial screening of titles and abstracts to identify potentially relevant studies. Then the same authors independently assessed the full texts of selected articles based on the predefined inclusion and exclusion criteria. Finally, any disagreements between the authors were resolved through discussions, and if a consensus could not be reached, a third author (SJK) was consulted to make the final decision. The study selection process began with the identification of 1994 records across multiple databases. Duplicate records (*n* = 1,588) were removed using reference management software. The remaining titles and abstracts were screened independently by two authors. Articles were categorized as “included,” “excluded,” or “maybe.” For “maybe” studies, full texts were retrieved and reviewed for eligibility. Final full-text assessments were conducted by both authors, with disagreements resolved through discussions or by consulting a third author (SJK). Ultimately, 26 studies were included in the final review. The selection process is illustrated in Figure 1 using a PRISMA flow diagram.

### 2.4 Step 4: charting data

The data charting process was conducted in multiple stages to ensure precision and consistency throughout the review. Initially, one author (HY) extracted relevant data from all included studies and organized it into a standardized spreadsheet format. Subsequently, a second author (XL) independently reviewed the extracted data to verify their completeness and accuracy. Any discrepancies identified during this verification process were discussed and resolved collaboratively by HY and XL to maintain data integrity. The extracted data encompassed several key aspects: study design, participant demographics, intervention characteristics, frequency and duration of sessions, total session count, and details regarding the integration of digital technology. Specifically, information was collected on the types of digital technology employed, feedback modalities provided to participants, and the context or setting in which the technology was used. In addition to digital technology, the analysis also focused on the musical components of the interventions. This included identifying the types of music activities (e.g., instrument playing, singing, rhythm exercises), the genres of music utilized, and any specific musical elements incorporated into the therapeutic processes. Graphical formats and summary tables were generated as needed to present the data in an accessible and interpretable manner. To ensure the accuracy of the summarized findings, HY and XL consistently referred to the original text of each included study during the charting process. All uncertainties or disagreements were addressed through discussions among the research team, ensuring that the final dataset was both comprehensive and systematically organized for subsequent analysis.

### 2.5 Step 5: collating, summarizing, and reporting the data

The stage of this scoping review was performed by the methodology framework outlined by Levac et al. (2010). A comprehensive descriptive synthesis of the data presented in the charting table was conducted by three reviewers (HY, XL, SJK), while qualitative content analysis techniques were applied by two reviewers (HY, SJK) to examine the scope and content of digital technology applications. The results from the summary and qualitative analysis were subsequently used to contextualize the results, specifically in relation to our research question. The analysis elucidated fundamental concepts, key themes, and patterns in music-based interventions using digital technology for individuals with brain injuries. This review highlights existing evidence while critically identifying gaps in the literature, providing a foundation for future research. The findings are detailed in the Results section, structured to address the research questions.

## 3 Results

This study included 26 research articles. All papers in this study were published between 2007 and 2024. Among them, four studies were published between 2007 and 2011, six between 2012 and 2018, and the remaining 16 studies were published within the last 6 years. Each study was analyzed based on intervention methods, participant characteristics, digital tools used, and study outcomes. General characteristics of the 26 studies, including research design, population, target goal area, intervention, and measurement available in Table 1.

**Table 1:** General characteristics of the study *N* = 26.

Authors (year)	Research design	Sample description: groups: N (Mean Age/Years)	Target area	Diagnosis, instructor, mode, frequency, duration	Procedure (steps, program)			Measurements
					Setup/ assessment	Training	Feedback/ evaluation	
Schneider et al. (2007)	RCT	I: *N* = 20 (58.1 ± 9.9) C (UT): *N* = 20 (54.5 ± 10.2)	Upper limb / Arm and hand	Stroke / NR / In / 5 times a week*3 weeks / 30 min	Diagnosis-based training	Rhythmic practice (drum pad), Unilateral to bilateral play (MIDI-piano)	Modulation	ARAT, APS, BBT, CMA, NHPT
Yoo (2009)	One-group pre-post	I: *N* = 3 (P1: 77, P2: 49, P3: 79)	Upper limb /Arm	Stroke / MT / In / 3 times a week*2 weeks / 35 min	Arm stretches, Muscle relaxation	MIDI drum and movement	Breathe	BI, FMA, MAS
Friedman et al. (2011)	Multiple baseline	I: *N* = 10 (NR)	Upper limb / Hand	Stroke / PT / In / 6*once / 2 min 59 s	Glove setup, Initial MusicGlove assessment	Playing (music and no-music trials)	Post-trial MusicGlove assessment,	BBT
Trobia et al. (2011)	One-group pre-post	I: *N* = 2 (P1: 68, P2: 39)	Upper limb /Arm	Stroke / NR / In / 3 times a week*4 weeks / NR	Observe (music + VR mirror)	Imitate (music-guided movements)	Practice (home exercises with videos/audio)	ARAT, FMA, VMIQ, MBEA
Cha et al. (2014)	RCT	I: *N* = 10 (59.8 ± 11.7) C: *N* = 10 (63.0 ± 14.1)	Walking	Stroke / MS / In / 5 times a week*6 weeks / 30 min	Baseline cadence measured	RAS with metronome/music; gait practice	NR	BBS, SS-QOL
Chong et al. (2014)	One-group pre-post	I: *N* = 8 (13)	Upper limb / Arm and hand	Brain damage(intracranial lesions) / MT / In / 3 times a week*4 weeks / 30 min	MIDI keyboard system setup	Melodic exercises; harmonic support by therapist	MIDI-recorded feedback	Grip and Pinch Strength Test, BBT, JHFT
Kirk et al. (2016)	Multiple case study	I: *N* = 3 (P1: 50, P2: 44, P3: 50)	Upper limb /Arm	Stroke / NR / In / 3 times a week*5 weeks / 30 min	Song selection	Cue setup, Drum pad Interaction	Rhythmic practice and movements	ALRT, SSI, Goniometer
Zondervan et al. (2016)	RCT	I: *N* = 9 (60), C: *N* = 8 (59)	Upper limb/hand	Stroke / Self / In / 3 times a week*3 weeks / 3 h per week	Device/software instruction	Self-guided MusicGlove exercises	Weekly follow-ups	BBT, MAL, NHPT, ARAT, FMA-UE, GDS
Silveira et al. (2018)	Clinical case report	I: *N* = 1 (74)	Upper limb / Hand	Stroke / MT / In / once a week*21 week / NR	ThumbJam programming, FES device setup	Focus on fingers, Independent play	Familiar tunes play	NHPT, Pinch dynamometer, Grip dynamometer
Street et al. (2018)	Pilot, RCT, cross-over	I: *N* = 6 (53.2), C: *N* = 5 (67.6),	Upper limb / Arm and hand	Stroke / Trained NMT / In / 2 times a week*6 weeks / 20-30 min	Movement/rhythm assessment; TIMP protocols	Upper limb exercises with music/iPads	Metronome precision	RAT, NHPT, Structured Interviews, Research diary
Chang and Lee (2019)	Comparati-ve experiment	I: *N* = 5/S = 2, HP = 3 (NR)	Walking	Stroke / NR / In / NR / NR	Target calibration, Initial participant setup	Stepping tracks (Music-beat targets)	Feedback rewards (score and visual rewards), Performance review	EEG, Gait information
Nikmaram et al. (2019)	RCT	S1: I: *N* = 7 (65.30 ± 12.70) C: *N* = 5 (66.40 ± 6.90) S2: I: *N* = 14 (68.71 ± 11.76) C: *N* = 14 (70.21 ± 14.29)	Upper limb /Arm	Stroke / NR / In / S1: I:TD = 15 (11–15) C:TD = 15 (13–15) S2: I:TD = 22 (7–40) C:TD = 16.5 (9–46) / 30 min	Setup and positioning	Scale practice, Specified positions for guided practice	Independent playing (3D sonification space)	ARAT, BBT, FMA, NHPT, SIS, Thumb localizing test
Wittwer et al. (2019)	Feasibility, single group repeated measure	I: *N* = 5 (54 ~ 74)	Walking	Progressive Supranuclear Palsy / PT / In / 3 times a week*8 weeks / 60 min	Warm-up activities	Gait training with RAC, personalized music, home practice	Home visits by physiotherapists	ACE-III, GDS, PPA, UPDRS, PSPRS
Segura et al. (2021)	RCT	I: *N* = 5 (52.6 ± 13.3) C(HP): *N* = 20 (10/M: 65.3, 10/F: 63.5)	Upper limb / Arm and hand	Stroke / MT / In / 3 times a week*10 weeks / 60 min	Percussion play	Tempo training, MIDI practice (Difficulty levels and Real-time feedback)	Piano evaluation, Performance tracking	ARAT, BBT, FMA, NHPT, Chedoke arm and hand activity inventory
Hankinson et al. (2022)	RCT	I: *N* = 10 (NR) C (UC): *N* = 12 (NR)	Upper Limb and Lower limb	Stroke / PT / In / 3 times a week*6 weeks / 20 min	Wireless IMU configuration, GotRhythm app initialization	Tracking movements, Tempo matching	Correction and realigned with the tempo, Feedback	FMA
Heyse et al. (2022)	RCT	I: *N* = 4 (NR) C (HP): *N* = 4 (NR)	Cognitive (Unilateral Spatial Neglect)	Stroke / PT / In / 3 times a week*2 weeks / 30 min	Play notes	Scale practice, Sequence memory, Free-to-play task	System adaptation (adjusts task difficulty), Therapist monitoring	CBS, TAP
Kantan et al. (2022)	RCT	I: *N* = 6 (NR)	Walking	Stroke / PT / In / NR / NR	Trunk stability feedback	Target synchronization (music tempo with target trajectory)	Rhythmic timing feedback, Music-gait alignment	SI, Fixed set of questions
Loria et al. (2022)	Multiple baseline	I: *N* = 28 (55.9 ± 12.3)	Upper limb /Arm	Stroke / NR / In / 3 times a week*3 weeks / 30 min	Rhythm cueing	Targeted movements, Movements focus training	Kinematics measurement	FMA, WMFT
Collimore et al. (2023)	One-group pre-post	I: *N* = 3 (70 ± 1)	Walking	Stroke / NR / In / 3 times a week*3 weeks/30 min	Sensor assessment	Adjust music tempo and beat, Sync scoring	Feedback adjustment, Adaptive looping	BOC, BPC, WS
Kogutek et al. (2023)	Multiple baseline	I: *N* = 10 (60.2 ± 8.6)	Upper limb /Arm	PD / PT / In / once / 30 min	Demonstration	Rhythmic measurement, Syncopation adjustment	Tempo complexity control	MHY Scale, SDM, Note count and mean note velocity
Sun et al. (2023)	Pilot/ protocol	I: *N* = 2 (NR)	Upper limb /Arm	Stroke / NR / NR / NR / NR	Setup (VR headset and controller), Introduction	Strike and feedback (Virtual Xylophone), Drumming (Virtual Drumset)	Drum positioning (Arm Extension and Movement), Assessment	NR
Zajac et al. (2023)	One-group pre-post	I: *N* = 23 (66.91 ± 8.78)	Walking	PD / NR / In / 5 times a week*4 weeks / 30 min	Calibration phase, Automatic rhythm cue settings	Tempo matching (Cadence-Based Synchronization), Adapt (Tempo adjustment)	Safety monitoring, User experience assessment	6MWT, 10MWT, FTSS, MDS-UPDRS Part III, PDQ
Impellizzeri et al. (2024)	Single-blind quasi-randomized controlled trial	I: *N* = 20 (62.35 ± 7.13) C: *N* = 20 (62.55 ± 9.59)	Cognitive/Executive	PD / Trained NMT / In / 3 times a week*8 weeks / 45 min	Warm-up with rhythmic music	CAREN scenarios; cool-down with rhythmic walking	NR	MoCA, HRSD, FAB
Segura et al. (2024)	RCT	I: *N* = 26 (64.2 ± 12.5) C: *N* = 32 (62.2 ± 12)	Upper limb / Arm and hand	Stroke / MT / In+Gp / 4 times a week(Gp3 + In1)*10 weeks / 60 min	Evaluation and customization	eMST app sessions, virtual group therapy	Telemonitoring and compliance tracking	ARAT, FMA-UE, CAHAI, BBT, NHPT, Grip strength dynamometer, BRIEF, SART, WMS-R, RAVLT, Fluency test in Spanish, BDI-II.
Tamir-Ostrover et al. (2024)	Pilot Open-Label Experimental Study	I: *N* = 3 (P1: 41, P2: 52, P3: 71)	Upper limb/hand	PD / NR / In / 6 week / 6 h total / NR	Pre-testing for dexterity	Piano training; supervised sessions, independent practice	Post-testing	BBT, MDS-UPDRS, PDQ-39, QDG
Tamplin et al. (2024)	Single-arm feasibility study	I: *N* = 28 (68)	Speech	PD / MT&SP / Gp / 12 weeks / 90 min	Eligibility screening	Breathing, speech, singing, social practice via Zoom	Follow-up assessments	DIS, DASS, PDQ-39, MDS-UPDRS

ABI, Acquired Brain Injury; ACE-III, Addenbrooke’s Cognitive Examination III; ALRT, Arms Length Reach Test; APS, Arm Paresis Score; ARAT, Action Research Arm Test; BDI-II, Beck Depression Inventory-II; BBS, Berg Balance Scale; BBT, Box and Block Test; BI, Barthel Index; BOC, Baseline Oxygen Consumption; BPC, Biomechanical and Physiological Changes; BRIEF, Behaviour Rating Inventory of Executive Function; C, Control Group; CAHAI, Chedoke Arm and Hand Activity Inventory; CBS, Catherine Bergego Scale; CMA, Computerized Movement Analysis; DASS, Depression Anxiety and Stress Scale; DIS, Dysarthria Impact Scale; EEG, Electroencephalography; FAB, Frontal Assessment Battery; FMA, Fugl-Meyer Assessment; FMA-UE, Fugl-Meyer Assessment for Upper-Extremity; FTSS, Five Times Sit-to-Stand Test; GDS, Geriatric Depression Scale; Gp, Group session; Gr, group session; Grip Strength Dynamometer; HP, Healthy Participants; HRSD, Hamilton Rating Scale for Depression; I, Intervention group; IMI, Intrinsic Motivation Inventory; In, individual session; M, Male; MAL, Motor Activity Log; MAS, Modified Ashworth Scale; MBEA, Montreal Battery Test of Evaluation of Amusia; MDS-UPDRS, Movement Disorder Society – Unified Parkinson’s Disease Rating Scale; MDS-UPDRS Part III, Unified Parkinson’s Disease Rating Scale Part III (Motor); MHY Scale, Modified Hoehn and Yahr Scale; MoCA, Montreal Cognitive Assessment; MS, Music Specialist; MT, Music Therapist; N, Number of Subjects; NA, not applicable; NHPT, Nine Hole Pegboard Test; NMT, Neurologic Music Therapist; NR, not reported; OT, Occupational Therapist; P, Participant; PD, Parkinson’s Disorder; PDQ, Parkinson’s Disease Questionnaire; PDQ-39, Parkinson’s Disease Questionnaire; PPA, Physiological Profile Assessment; PSPRS, Progressive Supranuclear Palsy Rating Scale; PT, Physical Therapist; QDG, Quantitative Digitography Test; RCT, Randomized Controlled Trial; RAVLT, Rey Auditory Verbal Learning Test; S, Stroke; S1, Site 1; S2, Site 2; SART, Sustained Attention to Response Task; SDM, Syncopation Density Metric; SI, Structured Interviews; SIS, Stroke Impact Scale; SP, Speech Pathologist; SSI, Semi-Structured Interview; TAP, Test of Attentional Performance; TD, Training Days; UC, Usual Care; UPDRS, Unified Parkinson’s Disease Rating Scale; UT, Usual Therapy; VMIQ, Vividness of Movement Imagery Questionnaire; VR, Virtual Reality; WMFT, Wolf Motor Function Test; WMS-R, Wechsler Memory Scale-Revised; WS, Walking Speed; 6MWT, 6 Minute Walk Test.

### 3.1 Functional focus and outcome measures of digital music-based interventions

**Figure 2:**
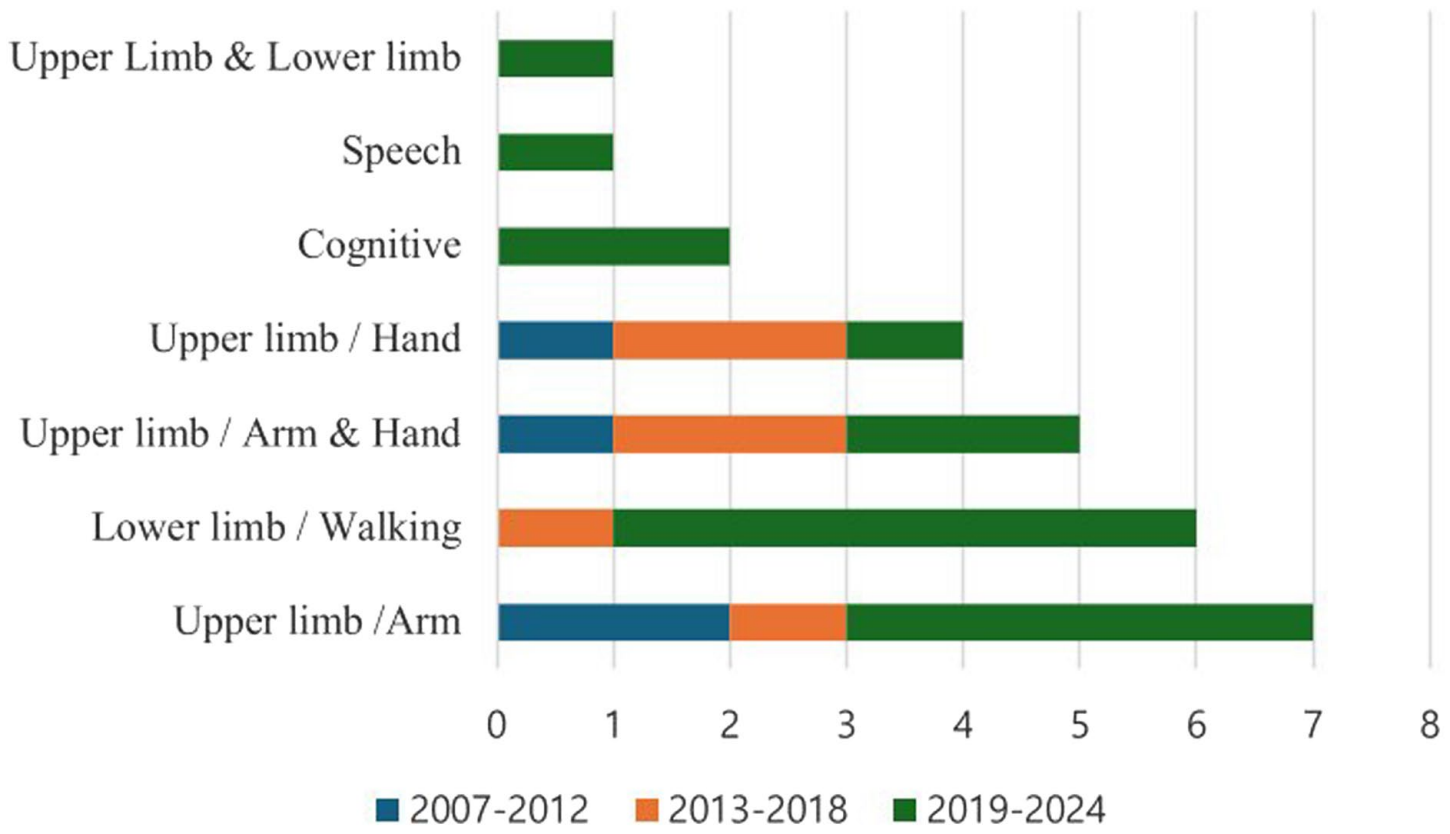
Analysis of the target area of the study.

The digital music-based interventions reviewed in this analysis targeted three main functional areas: motor functions (including upper limbs, lower limbs, hand functions, and combined arm and hand functions and combined upper and lower limb functions), cognitive functions, speech rehabilitation. Seven studies (26.9%) focused on upper limb/arm functions, six studies (23.1%) on lower limb/walking functions, five studies (19.2%) on combined upper limb/arm and hand functions, and four studies (15.4%) specifically targeted hand functions. One study (3.8%) addressed both upper and lower limb functions. Additionally, two studies (7.7%) focused on cognitive functions, and one study (3.8%) targeted speech rehabilitation (see Figure 2).

Outcome measures across the reviewed studies were categorized into four domains: motor function, cognitive outcomes, and quality of life. Motor function improvements were consistently reported through assessments such as the Action Research Arm Test (ARAT) (Schneider et al., 2007; Trobia et al., 2011; Nikmaram et al., 2019; Segura et al., 2021; Segura et al., 2024), Fugl-Meyer Assessment (FMA-UE) (Trobia et al., 2011; Yoo, 2009; Zondervan et al., 2016; Nikmaram et al., 2019; Segura et al., 2021; Segura et al., 2024), BBT (Chong et al., 2014; Schneider et al., 2007; Friedman et al., 2011; Nikmaram et al., 2019; Segura et al., 2021; Segura et al., 2024; Zondervan et al., 2016) and gait analysis tools like the GAITRite system (Cha et al., 2014; Wittwer et al., 2019), with significant advancements in paretic limb coordination and acceleration. Cognitive outcomes evaluated using tools like Catherine Bergego Scale (Heyse et al., 2022), the Montreal Cognitive Assessment (MoCA) and Stroop Test, demonstrated notable gains in memory, executive function, and mental engagement, particularly in interventions utilizing immersive digital tools and rhythmic tasks (Heyse et al., 2022; Impellizzeri et al., 2024). Emotional well-being, assessed through scales such as Geriatric Depression Scale (GDS) (Wittwer et al., 2019; Zondervan et al., 2016), showed high motivation and emotional engagement, especially in interactive music-based interventions like digital drum pads. Quality of life metrics, including the Stroke Impact Scale (SIS) (Nikmaram et al., 2019) and Parkinson’s Disease Questionnaire (Tamir-Ostrover et al., 2024; Tamplin et al., 2024; Zajac et al., 2023), revealed significant improvements in mobility, endurance, and daily functioning, with interventions leveraging gamification and immersive environments demonstrating enhanced adherence and patient satisfaction. Collectively, these studies highlight the efficacy of music-based digital interventions in addressing multidimensional rehabilitation goals through quantitative methodologies.

### 3.2 Categorization and evolution of digital music-based rehabilitation tools

The digital music-based interventions in this analysis were grouped into four primary types, each covering a distinct aspect of rehabilitation technology: (1) mobile and app-based music rehabilitation tools, which encompass software applications designed for easy use, portability, and interactive engagement. They allow participants to engage in musical exercises through mobile devices or tablets, such as the eMST app (Segura et al., 2024), proprietary mobile applications (Zajac et al., 2023), and gamified music tools (Street et al., 2018; Tamplin et al., 2024); (2) sensor-integrated music feedback systems, which involve using wearable sensors (e.g., accelerometers, wireless wearable sensors) that provide real-time feedback during musical activities, facilitating motor coordination and synchronization Examples include the GotRhythm system (Hankinson et al., 2022), SONATA (Loria et al., 2022), and MIDI-integrated music systems (Chong et al., 2014; Nikmaram et al., 2019); (3) VR/Augmented reality (AR) music therapies, which utilize immersive environments to enhance engagement, combining music with visual or motion-based interaction. Examples include VR treadmill systems (Impellizzeri et al., 2024), MR goggles (Chang and Lee, 2019), and VR musical instruments like xylophones and drums (Sun et al., 2023); and (4) modified/electronic drums and rhythm instruments, which include specially adapted musical instruments like electronic drum sets and MIDI components that are used to support rhythmic training and motor skills improvement (e.g., Schneider et al., 2007; Yoo, 2009) (see Table 2). These technology tools highlight a shift toward personalized and interactive rehabilitation, particularly for motor functions. Upper limb rehabilitation was the most targeted area, with 17 studies focusing on improving daily motor skills. While most studies focused on upper limb functions (*n* = 17), only one study addressed interventions for both upper and lower limbs (Hankinson et al., 2022), indicating a potential area for future exploration. Overall, cognitive function was represented less than motor function.

**Table 2:** Digital utilization by type of music-based rehabilitation *N* = 26.

Authors (year)	Types of interventions	Digital utilization
Schneider et al. (2007)	Modified/Electronic Drums and Rhythm Instruments	Electronic drum set consisting of 8 percussion pads, MIDI-piano
Yoo (2009)	Modified/Electronic Drums and Rhythm Instruments	MIDI drum (four drums), Roland TD-5 Percussion Sound Module, Roland KC-100 keyboard amplifier, Midiman Portman 2 × 4 MIDI interface box
Friedman et al. (2011)	Mobile and App-Based Music Rehabilitation Tools	MusicGlove/Gloves + USBController, Frets on Fire (FOF)/open source computer game
Trobia et al. (2011)	VR/AR Music Therapies	VR Mirror, Back-projected horizontal scree, Movement tracking sensors
Cha et al. (2014)	Sensor-Integrated Music Feedback Systems	MIDI Cubase Musical Instrument Digital Interface (MIDI) Program, GAITRite system, metronome-integrated music, KM Player, synthesizer keyboard.
Chong et al. (2014)	Sensor-Integrated Music Feedback Systems	MIDI-compatible electronic keyboard (YAMAHA DGX-230), MIDI interface (Infrasonic AMON), MIDI sequencing program (Cubase 6), Laptop (LG Xnote P33).
Kirk et al. (2016)	Mobile and App-Based Music Rehabilitation Tools	Digital drum pads iPad App-open frameworks iOS release v 0.8.4 and Xcode 6.3 IDE
Zondervan et al. (2016)	Mobile and App-Based Music Rehabilitation Tools	MusicGlove device with embedded sensors, Laptop with pre-installed MusicGlove software.
Silveira et al. (2018)	Mobile and App-Based Music Rehabilitation Tools	Verity Neurotrac, ThumbJam/iOS music instrument application, iPads
Street et al. (2018)	Mobile and App-Based Music Rehabilitation Tools	Touchscreen plectrum, Garageband and Thumbjam/iOS music apps, iPads, Metronome, Arpiec.
Chang and Lee (2019)	Mobile and App-Based Music Rehabilitation Tools	MR music rehabilitation system, Android application, MR goggles (HoloLens), inertial measurement unit (IMU) sensors (Notch), tablet, wet-electrode EEG cap (NuAmps)
Nikmaram et al. (2019)	Sensor-Integrated Music Feedback Systems	Xsens inertial sensors, Leapmotion controller, Sonification (changes in musical pitch) of movements
Wittwer et al. (2019)	Modified/Electronic Drums and Rhythm Instruments	Handheld digital music player (SanDisk Clip Sport), Portable speaker (Ultimate Ears), Commercial software (Tempo Magic Pro), GAITRite mat.
Segura et al. (2021)	Mobile and App-Based Music Rehabilitation Tools	eMST Tablet-based application, Percussion exercises, Video tutorials, MIDI piano, Electronic drums, Gamification elements and remote monitoring
Hankinson et al. (2022)	Sensor-Integrated Music Feedback Systems	GotRhythm App, High-resolution recording of motor performance, Wireless wearable sensors (IMUs), Real-time auditory feedback
Heyse et al. (2022)	VR/AR Music Therapies	Virtual Reality system/using Unity3D, Dashboard component/using vue.js, Unity3D, Oculus Quest 2 Head-Mounted Display, PostgreSQL database, Python script, ZeroMQ message bus, Gaming laptop
Kantan et al. (2022)	Sensor-Integrated Music Feedback Systems	Music Generation and Biofeedback Control Software, Sensor Interface and Data Processing, Software Components-JUCE, FAUST, Custom Type-1 MIDI File Format, Hardware Components-M5Stack Grey Microcontrollers, Arduino ID
Loria et al. (2022)	Sensor-Integrated Music Feedback Systems	Sonification Arm Training Apparatus/SONATA, Digital Metronome Device, GENEActiv Action Accelerometer, digital auditory devices/Yamaha CP40 Stage Piano and DTX Drums
Collimore et al. (2023)	Mobile and App-Based Music Rehabilitation Tools	Autonomous Rehabilitation System, Automated Treatment Algorithms, Bone Conduction Headphones, Wireless Inertial Sensors, Motion Analysis System/Qualisys 18-camera system, Biomechanics Software/Visual3D, Gait Event Detection Algorithm
Kogutek et al. (2023)	Mobile and App-Based Music Rehabilitation Tools	Humdrum Toolkit, Logic Pro MIDI, MATLAB, Metronome
Sun et al. (2023)	VR/AR Music Therapies	VR Game/Using PICO4: Xylophone Play Mode and Drums Play Mode, PICO4 VR Headset, Multi-Sensory Feedback, Screen Mirroring Capability
Zajac et al. (2023)	Mobile and App-Based Music Rehabilitation Tools	Proprietary Software Mobile Application, Locked Touchscreen Device with Android OS, Foot Sensors, Headset, Charging Equipment
Impellizzeri et al. (2024)	VR/AR Music Therapies	CAREN System with VR treadmill integration, 5.1 surround sound system, acoustic instruments, Metronome, tambourines, maracas, Immersive virtual scenarios.
Segura et al. (2024)	Mobile and App-Based Music Rehabilitation Tools	eMST app for gamification and feedback, Percussion instruments, Zoom platform, tracking/telemonitoring software.
Tamir-Ostrover et al. (2024)	Sensor-Integrated Music Feedback Systems	Instrumented Nine-Hole Peg Test with Arduino, Custom MIDI Keyboard.
Tamplin et al. (2024)	Mobile and App-Based Music Rehabilitation Tools	Proprietary software (Redenlab Online) for voice/speech recording, Locked USB headsets, Zoom, Canvas online repository.

AR, Augmented Reality; eMST, electronic Music Synchronization Therapy; EEG, Electroencephalography; FAUST, Functional Audio Stream Processing; IMU, Inertial Measurement Unit; MATLAB, Matrix Laboratory; MIDI, Musical Instrument Digital Interface; MR, Mixed Reality; VR, Virtual Reality.

Chronologically, the interventions have evolved. Early studies (2007–2011) emphasized rhythmic and MIDI-based tools (Schneider et al., 2007; Yoo, 2009), demonstrating significant improvements in motor control measures like the ARAT. Six studies conducted between 2012 and 2018 applied instrumental playing for upper limb rehabilitation. These utilized the MIDI-based tools, musical sonification training system (Trobia et al., 2011) and VR instrumental playing (Kirk et al., 2016). Interventions utilizing MIDI have been consistently employed (Cha et al., 2014; Chong et al., 2014). Additionally, technology-based devices such as the MusicGlove (Zondervan et al., 2016) and app-based programs (Silveira et al., 2018; Street et al., 2018) have also been applied. From 2019 onwards, there was a clear shift toward integrating adaptive feedback systems (Hankinson et al., 2022; Tamir-Ostrover et al., 2024), VR/AR technologies (Heyse et al., 2022; Impellizzeri et al., 2024; Sun et al., 2023), and gamification (Segura et al., 2024; Zajac et al., 2023), resulting in substantial improvements in gait, upper limb function, and quality of life.

Musical elements were carefully tailored to therapeutic goals, with rhythm and melody being the most frequently utilized for motor rehabilitation (Street et al., 2018; Wittwer et al., 2019). Instruments like digital pianos, percussion pads, and virtual instruments enabled precise control and customization (Chong et al., 2014; Silveira et al., 2018). For cognitive interventions, elements like harmony, dynamic tones, and multimodal feedback were more prominent, fostering engagement and memory enhancement (Heyse et al., 2022; Impellizzeri et al., 2024).

### 3.3 Analysis of technological tools in music-based interventions

This scoping review examined the role of digital technology in music-based interventions for individuals with brain injuries. Of the 26 studies, most targeted physical function outcomes, with interventions categorized by technological setup, training methods, and feedback mechanisms (see intervention part in Table 1). The initial setup phase, which involved preparing and familiarizing participants with the digital tools, included different technologies: advanced VR systems and holographic displays, MIDI controllers, and MusicGlove. These technologies played a critical role in helping participants acclimate to the technology, ensuring comfort with the technology and creating an interactive foundation for the intervention. These tools provided an interactive foundation and ensured participants’ familiarity with the systems. For instance, VR headsets (Sun et al., 2023) created immersive environments for upper-limb tasks, while MIDI controllers enabled precise motor-skill training with rhythmic exercises (Yoo, 2009). Technologies like the CAREN system (Impellizzeri et al., 2024) were particularly notable for combining VR with music-based scenarios, offering comprehensive environments for cognitive and motor rehabilitation.

The training phase showcased a variety of methods designed to enhance motor skills via musical behaviors. Rhythmic synchronization was commonly emphasized, with studies like Schneider et al. (2007) and Kirk et al. (2016) utilizing drum pads and digital percussion to support motor control. MIDI-based exercises were also used; Friedman et al. (2011) used the MusicGlove to provide real-time feedback on finger movements, and Nikmaram et al. (2019) focused on scale practice using MIDI controllers to improve stroke recovery metrics. Virtual instrument interactions were incorporated as well, with studies such as Sun et al. (2023) using virtual xylophones and drum sets to encourage tempo matching and movement synchronization. The integration of mobile apps and gamified platforms (Segura et al., 2021; Zajac et al., 2023) further exemplified the flexibility of digital tools in delivering personalized and engaging interventions.

Feedback and evaluation played a critical role in delivering these interventions. Many studies employed adaptive feedback mechanisms to adjust the therapy based on real-time participant performance. For instance, Segura et al. (2021) and Street et al. (2018) used dynamic feedback systems to tailor the difficulty of their respective interventions, ensuring personalized experiences that sustained participant engagement and improved accuracy of movement. Additionally, performance tracking allowed for continuous assessment of progress, as seen in studies by Collimore et al. (2023) and Kantan et al. (2022) that incorporated gait and rhythm tracking to optimize outcomes. Additionally, performance monitoring tools like MIDI-based analysis (Chong et al., 2014) and gamified feedback systems (Chang and Lee, 2019) facilitated continuous assessment, enabling substantial improvements in motor skills, engagement, and overall therapeutic effectiveness. These feedback mechanisms, integral to most interventions, highlighted the importance of real-time responsiveness in achieving meaningful rehabilitation outcomes (see Table 3).

**Table 3:** Analysis of digital technology and music usage by target area N = 26.

Target area	Authors (year)	Types of Interventions	Music usage	Musical elements
Upper limb /Arm	Yoo (2009)	Modified/Electronic Drums and Rhythm Instruments	Instrumental Performance	Participant: MIDI Drum Playing-Beat and Timbre (instrument selection) MT: Live Music-Rhythm and Scale and Volume and Harmony, Melody (preferences and familiar songs)
	Trobia et al. (2011)	Modified/Electronic Drums and Rhythm Instruments	Background Music	Track Selection - Tempo Rhythm and Tonality and Preferences
	Kirk et al. (2016)	Mobile and App-Based Music Rehabilitation Tools	Instrumental Performance	Rhythm/Melody (adaptation of favorite songs)
	Nikmaram et al. (2019)	VR/AR Music Therapies	Instrumental Performance	Melodic and Scales
	Loria et al. (2022)	Mobile and App-Based Music Rehabilitation Tools	Rhythmic signals	Rhythmic
	Kogutek et al. (2023)	Mobile and App-Based Music Rehabilitation Tools	Instrumental Performance	Rhythm and Melody and Beat
	Sun et al. (2023)	Sensor-Integrated Music Feedback Systems	Instrumental Performance	Rhythm and Timbre (instrumental choice)
Upper limb / Hand	Friedman et al. (2011)	VR/AR Music Therapies	Instrumental Performance	Rhythm and Melody and Favorite Songs
	Zondervan et al. (2016)	Mobile and App-Based Music Rehabilitation Tools	Instrumental Performance	Rhythm and Melody, Timbre, Interactive Engagement
	Silveira et al. (2018)	Mobile and App-Based Music Rehabilitation Tools	Instrumental Performance	Timbre (instrument selection) and Melody and Familiar Songs
	Tamir-Ostrover et al. (2024)	Sensor-Integrated Music Feedback Systems	Instrumental Performance	Rhythm and Melody (piano repertory), Tempo and timing customization
Upper limb / Arm and hand	Schneider et al. (2007)	Sensor-Integrated Music Feedback Systems	Instrumental Performance	Beat and Melody and Scale
	Chong et al. (2014)	Sensor-Integrated Music Feedback Systems	Instrumental Performance	Rhythm and Melody (simple to complex patterns), Harmonic Accompaniment
	Street et al. (2018)	Mobile and App-Based Music Rehabilitation Tools	Instrumental Performance	Rhythm, Timbre, and Melody
	Segura et al. (2021)	Sensor-Integrated Music Feedback Systems	Instrumental Performance	Melodic/Rhythm and Timbre (instrument selection)
	Segura et al. (2024)	Mobile and App-Based Music Rehabilitation Tools	Instrumental Performance	Rhythm (keyboard and percussion exercises), Gamification elements (interactive music rhythms).
Lower limb / Walking	Cha et al. (2014)	Sensor-Integrated Music Feedback Systems	Rhythmic Signals	Rhythm and Timbre, Participant-preferred tracks (melody, tempo adjustments)
	Chang and Lee (2019)	Mobile and App-Based Music Rehabilitation Tools	Background Music	Beat and Melody
	Wittwer et al. (2019)	Modified/Electronic Drums and Rhythm Instruments	Background Music	Rhythm (personalized tempo-adjusted tracks), Timbre (portable speakers)
	Kantan et al. (2022)	Mobile and App-Based Music Rehabilitation Tools	Background Music	Beat and Melody (sound and volume changes)
	Collimore et al. (2023)	Mobile and App-Based Music Rehabilitation Tools	Background Music	Beat and Melody (volume change)
	Zajac et al. (2023)	VR/AR Music Therapies	Background Music	Beat and Melody
Cognitive	Heyse et al. (2022)	Mobile and App-Based Music Rehabilitation Tools	Instrumental Performance	Beat and Melody and Scales
	Impellizzeri et al. (2024)	VR/AR Music Therapies	Instrumental Performance and Rhythmic Signals	Participant: MIDI Drum Playing-Beat and Timbre (instrument selection), Therapist: Live Music (rhythm, scale, melody preferences).
Speech	Tamplin et al. (2024)	Mobile and App-Based Music Rehabilitation Tools	Vocal Performance	Rhythm, Melody, Timbre, Pitch, Volume (adaptive)
Upper Limb and Lower limb	Hankinson et al. (2022)	Mobile and App-Based Music Rehabilitation Tools	Background Music	Beat and Rhythm-Preferred Music

AR, Augmented Reality; MIDI, Musical Instrument Digital Interface; MT, Music Therapist; VR, Virtual Reality.

### 3.4 Use of digital tools in assessment and personalization

This review of 26 studies demonstrates the integration of digital tools in music therapy, showcasing their varied applications in both assessment and intervention (see Table 4). Among these, 11 studies used digital evaluation tools before and after their intervention. By comparing pre-and post-intervention data, these studies offered objective evidence of their intervention’s effectiveness. For instance, in studies involving rhythmic auditory stimulation (RAS) for gait rehabilitation, functional improvements were measured by tracking changes in step cadence and rhythm throughout the intervention process. This method not only reinforced the objectivity of the outcomes but also provided valuable insights for developing personalized follow-up treatment plans.

**Table 4:** Usage of digital tools in pre- and post-assessment *N* = 26.

Authors (year)	Transformation/products	Activity/technique	Use of digital tools assessment	
			Pre	Post
Schneider et al. (2007)	Modified/Electronic drums and electronic rhythm instruments	Playing	Y	Y
Yoo (2009)	Technology Combination/VR mirror and projector and Movement tracking sensors	TIMP	Y	Y
Friedman et al. (2011)	Development/Electronic drums and iOS app	TIMP	N	Y
Trobia et al. (2011)	Development/Musical sonification training system (Xsens inertial sensors and Leapmotion controller and Sonification of movements)	Listening	N	N
Cha et al. (2014)	Technology Combination / MIDI Cuebase and GAITRite system and Metronome-integrated music and Synthesizer keyboard.	RAS	Y	Y
Chong et al. (2014)	Technology Combination / MIDI Keyboard (YAMAHA DGX-230) and MIDI Interface and Sequencing Software (Cubase 6).	Playing	Y	Y
Kirk et al. (2016)	Technology Combination/Gear VR and classical audio and egocentric 180° 3D video clips	Playing	N	N
Zondervan et al. (2016)	Development / MusicGlove: Proprietary device with embedded sensors and MusicGlove software for auditory feedback.	Playing	N	N
Silveira et al. (2018)	Development/Arm training device with integrated digital metronome	Playing	N	N
Street et al. (2018)	Technology Combination / Touchscreen Plectrum and Garageband and Thumbjam/iOS Music Instrument Application and iPads.	TIMP	N	N
Chang and Lee (2019)	Technology Combination/Logic Pro MIDI improvisation with MATLAB integration	RAS	NR	NR
Nikmaram et al. (2019)	Development/Virtual reality performance environment using immersive experience controllers and VR devices	Playing	N	N
Wittwer et al. (2019)	Technology Combination / Digital Music Player and Tempo Magic Pro Software and GAITRite mat for gait analysis.	RAS	Y	Y
Segura et al. (2021)	Development/Customizable musical biofeedback (wireless wearable sensor system and open-source)	Playing	N	N
Hankinson et al. (2022)	Modified/Electronic drums with piano sound output and MIDI keyboards	RAS	N	N
Heyse et al. (2022)	Development/MusicGlove and FOF open source computer software	Playing	N	N
Kantan et al. (2022)	Technology Combination/Touchscreen plectrum and GarageBand, ThumbJam/iOS app	Listening	N	N
Loria et al. (2022)	Development/eMST program development app for online therapy and MIDI piano and electronic percussion instruments	Playing	N	N
Collimore et al. (2023)	Technology Combination/FES + ThumbJam/iOS app	TIMP	Y	Y
Kogutek et al. (2023)	Development/A multi-sensory VR tool for USN (patient xylophone exercises and therapist dashboard for session control)	RAS	Y	Y
Sun et al. (2023)	Development/VR-MAT system for bilateral drumming with Logic Pro X system and electronic instruments	Playing	NR	NR
Zajac et al. (2023)	Development/Music training software for iOS system	RAS	Y	Y
Impellizzeri et al. (2024)	Development / CAREN System: VR treadmill and Live Music Integration and Immersive Virtual Scenarios and Metronome cues.	RAS, TIMP	Y	Y
Segura et al. (2024)	Development / eMST App and Percussion Instruments with digital patterns and Zoom platform for telemonitoring.	Playing	N	N
Tamir-Ostrover et al. (2024)	Development / Instrumented Nine-Hole Peg Test with Arduino integration and Custom MIDI Keyboard.	Playing	Y	Y
Tamplin et al. (2024)	Development / Proprietary Software (Redenlab Online™) and USB-connected headsets and Zoom for music streaming.	Singing	Y	Y

AR, Augmented Reality; FES, Functional Electrical Stimulation; FOF, Free and Open-Source Framework; MIDI, Musical Instrument Digital Interface; MR, Mixed Reality; N, No; NR, Not Reported; RAS, Rhythmic Auditory Stimulation; TIMP, Therapeutic Instrumental Music Performance; USN, Unilateral Spatial Neglect; VR, Virtual Reality; VR-MAT, Virtual Reality Music-Assisted Therapy; Y, Yes.

In contrast, 13 studies focused on the use of digital tools to personalize interventions, rather than providing real-time evaluation and feedback. These tools were used to tailor treatment protocols to meet the individual needs of patients. For example, motion tracking sensors combined with music feedback systems were primarily employed to design personalized motor training programs, allowing patients to engage in rehabilitation exercises specifically aligned with their functional abilities. Although these studies did not incorporate continuous real-time evaluation, the personalized interventions facilitated by digital tools played a key role in improving patient outcomes. The remaining two studies (Chang and Lee, 2019; Sun et al., 2023) did not report the use of digital tools in the assessment process.

## 4 Discussion

This review synthesized findings from 26 research articles on digital music interventions for individuals with neurological conditions, revealing varied research designs, intervention strategies, and technological applications. The studies primarily focused on brain injuries, applying rigorous methodologies to assess motor, cognitive, and emotional outcomes.

This review identified an evolution in intervention methodologies, reflecting an expanding scope and growing interest in the use of digital music as technology advanced. Early research (2007–2011) largely focused on rhythmic and MIDI-based interventions, primarily improving motor functions, such as tapping frequency and motor control. Electronic instrument playing interventions were reported between 2014 and 2018, and from 2019 onwards, the scope of interventions expanded to include more sophisticated tools like VR and holographic exercises, which enhanced not only motor functions but also cognitive engagement. From 2020 onwards, there was a notable shift toward even more advanced approaches, incorporating VR headsets, adaptive systems, and personalized feedback mechanisms. These recent developments have extended the impact of digital music interventions to areas such as gait improvement, upper limb function, and overall quality of life. However, the integration of these complex tools also introduced new challenges, including overcoming device complexity and ensuring user comfort. While immersive VR and AR environments hold significant promise, ongoing efforts are required to refine these technologies to further enhance user experience and maximize therapeutic efficacy.

The studies reviewed also highlighted both advantages and limitations in the use of digital tools for assessment and intervention. Digital tools for assessment, such as pre-and post-intervention evaluations, allowed for objective, quantitative measures of patient progress, enhancing the precision of motor, cognitive, and emotional outcome tracking. However, there were notable limitations, including the complexity of some devices, which reduced accessibility for elderly patients or those with severe impairments. Additionally, inconsistent assessment integration, with a significant number of studies not employing structured pre-or post-evaluations, indicates a need for standardized assessment approaches. Therefore, while the use of digital assessments presents opportunities for precision, usability challenges must be addressed to improve broader application in diverse populations.

The majority of digital music-based interventions reviewed in this study were focused on motor function rehabilitation, particularly targeting upper limb recovery. This is consistent with the high demand for restoring motor abilities in patients with neurological impairments, reflecting the critical role of upper limb mobility in daily living activities. The focus on upper limbs also meant that interventions commonly involved activities like musical instrument playing, as these tasks are particularly effective in encouraging precise, repetitive movements that are essential for motor re-learning and neuroplasticity. Our findings align with those of Altenmüller and James (2020), which also emphasized the significant benefits of using musical activities to enhance motor re-learning and foster neuroplasticity in patients with upper limb impairments. The studies reviewed in this paper have similarly highlighted the effectiveness of music-based upper limb rehabilitation in improving outcomes such as hand dexterity and coordination. While this approach demonstrates strong efficacy in enhancing motor function, it may inadvertently overlook other important therapeutic goals, such as emotional well-being or cognitive development, suggesting a need for more balanced intervention designs in future research.

A distinctive strength of the digital music-based interventions reviewed in this study lies in their ability to be personalized, which plays a crucial role in rehabilitation contexts. Personalized interventions allow therapy to be tailored to each patient’s unique needs, capabilities, and progress, thereby maximizing therapeutic outcomes. This personalization is accomplished through adaptive feedback mechanisms that adjust the intensity, difficulty, or type of musical activity in real time based on the patient’s performance. Our findings are consistent with those of Lai-Tan et al. (2023), which highlighted the importance of personalized musical elements—such as rhythm, melody, and tempo—in meeting patients’ specific therapeutic needs. The studies reviewed further emphasized the use of dynamic feedback to personalize music exercises, which helps sustain patient motivation and engagement, both of which are vital for effective rehabilitation. Rhythm is frequently employed to aid motor synchronization, while melody is used to enhance cognitive engagement, ensuring a balanced approach that addresses physical as well as emotional needs. This dual focus is particularly important for maintaining adherence in long-term rehabilitation programs.

When compared to other digital interventions, such as those involving VR for cognitive rehabilitation, the personalized nature of digital music-based interventions presents distinct advantages and challenges. The VR-based approaches, as described in Quan et al. (2024), leverage highly immersive environments to improve memory, attention, motor function, and social skills. For conditions like stroke and TBI, immersive VR experiences are particularly beneficial in enhancing patient focus and improving therapeutic outcomes through intensive engagement. However, these highly immersive systems require substantial technological infrastructure and expertise, which can limit their feasibility, especially in home-based or resource-constrained environments. In contrast, for conditions like Alzheimer’s disease in which cognitive demands must be carefully managed, non-immersive or semi-immersive VR may offer a more suitable balance by providing beneficial cognitive stimulation without overwhelming cognitive load. On the other hand, digital music-based interventions present inherent flexibility with fewer technological requirements, making them accessible across a wider range of settings. These interventions foster emotional engagement through musical elements, which is crucial for sustaining long-term motivation. However, they may not provide the same level of immersive, multisensory input as VR, which is often essential for social skills training and deep cognitive engagement. To maximize the benefits of both approaches, future research should explore hybrid models that integrate personalized music elements within immersive VR environments, thereby combining the adaptability and emotional resonance of music with the focused, multisensory engagement of VR. Such integrated solutions could lead to more effective, comprehensive, and patient-centered rehabilitation strategies.

The intervention process in these studies typically consisted of distinct stages, each characterized by specific technological and methodological requirements. The setup phase was essential for familiarizing patients with the digital tools, ensuring comfort, and preparing them to engage effectively with the technology. This phase often employed simpler tools, such as MIDI controllers or MusicGlove, to gradually introduce participants to the intervention environment, thereby minimizing anxiety and promoting ease of use. During the training phase, a variety of methods, including drumming exercises and guided movement feedback using VR, were utilized to enhance both motor and cognitive functions. A key feature across these interventions was the use of adaptive feedback to tailor the intensity of the exercises to each patient’s capabilities, helping to maintain motivation and engagement and ultimately improve motor performance. Finally, the evaluation and feedback phase played an important role in monitoring progress and refining future treatment protocols. By incorporating dynamic feedback mechanisms, participants received continuous input on their performance, which contributed to improved accuracy and sustained progress in motor rehabilitation. These structured phases collectively underscore the importance of careful intervention planning and the use of adaptive, feedback-driven approaches to fully leverage the therapeutic benefits of digital music-based tools.

In addition to the structured intervention process, this review also explored the distinct characteristics and implications of the four primary categories of digital music-based interventions (i.e., mobile and app-based tools, sensor-integrated feedback systems, VR/AR music therapies, and modified/electronic drums and rhythm instruments). Each category revealed unique advantages and limitations from both music therapy and rehabilitation perspectives. For example, from a music therapy standpoint, VR/AR tools provide an immersive experience that facilitates deep engagement in therapeutic processes, allowing patients to interact with music in a virtual environment that can enhance both cognitive and emotional involvement. However, the complexity and cost associated with VR systems can present significant challenges, especially in resource-limited settings. In contrast, sensor-integrated systems offer precise, real-time feedback that is invaluable for monitoring and adjusting motor rehabilitation. However, these systems may limit the emotional and creative aspects of music engagement, which are often important components of music therapy.

These findings indicate that each type of intervention offers distinct strengths depending on the target therapeutic goals and the specific needs of the patient. However, there is also a pressing need to optimize these technologies to enhance their adaptability and overcome current limitations. This could involve combining elements from different categories to create more holistic and comprehensive interventions that address not only motor function but also emotional and cognitive rehabilitation needs. However, this study primarily focused on digital-based music interventions for ABI patients, leading to the inclusion of research predominantly centered on physical or cognitive functions. Consequently, studies utilizing music for psychotherapy or counseling approaches were excluded. Future research should explore the integrative role of digital technology and music in alleviating symptoms of ABI patients through detailed analysis of digital-based music psychotherapy interventions.

Future research should investigate the integration of adaptive feedback mechanisms and advanced multimodal technologies, such as VR combined with biofeedback, to optimize rehabilitation processes. Moreover, it is essential to standardize assessment protocols to evaluate the consistency of outcomes across diverse populations and explore the scalability of these interventions for broader accessibility. To achieve these goals, the involvement of music therapist is crucial, as their expertise ensures the alignment of therapeutic objectives with technological innovations, thereby enhancing the personalization and effectiveness of rehabilitation strategies.
