# Supplementary material for: Music-based interventions using digital technology for individuals with acquired brain injuries: a scoping review
Source: Front Psychol. 2025 Feb 5;16:1532925. doi: 10.3389/fpsyg.2025.1532925 (PMC11838020; doi:10.3389/fpsyg.2025.1532925)
Supplement: Supplementary file 1 [file Data_Sheet_1.pdf]

Supplementary Materials

1. PubMed Search Strategy

("acquired brain injury" OR "stroke" OR "parkinson's") AND ("music" OR "instrument" OR "playing" OR “rhythm”)AND ("digital" OR "online" OR "AI" OR "artificial intelligence" OR "VR" OR "virtual reality" OR "MR" OR "mixed reality" OR "metaverse")

1. acquired brain injury (3,273)
2. stroke (342,767)
3. parkinson's (124,614)
4. #1 OR #2 OR #3 (46,641)
5. music (24,613)
6. instrument (155,805)
7. playing (58,610)
8. rhythm (99,136)
9. #6 OR #7 OR #8 OR #9 (333,870)
10. digital (215,185)
11. online (288,658)
12. AI (73,316)
13. artificial intelligence (64,680)
14. VR (15,747)
15. virtual reality (21,713)
16. MR (187,900)
17. mixed reality (1,374)
18. metaverse (621)
19. #11 OR #12 OR #13 OR #14 OR #15 OR #16 OR #17 OR #18 OR #19 (804,032)
20. #5 AND #10 AND #20 (390)

2. CINAHL Search Strategy

("acquired brain injury" OR "stroke" OR "parkinson's") AND ("music" OR "instrument" OR "playing" OR “rhythm”)AND ("digital" OR "online" OR "AI" OR "artificial intelligence" OR "VR" OR "virtual reality" OR "MR" OR "mixed reality" OR "metaverse")

1. acquired brain injury (2,113)
2. stroke (87,623)
3. parkinson's (36,948)
4. #1 OR #2 OR #3 (200,870)
5. music (21,732)
6. instrument (162,299)
7. playing (19,826)
8. rhythm (25,624)
9. #5 OR #6 OR #7 OR #8 (163,922)
10. digital (63,389)
11. online (115,831)
12. AI (18,011)
13. artificial intelligence (20,289)
14. VR (7,528)
15. virtual reality (10,575)
16. MR (29,479)
17. mixed reality (813)
18. metaverse (211)
19. #10 OR #11 OR #12 OR #13 OR #14 OR #15 OR #16 OR #17 OR #18 (239,104)
20. #4 AND #9 AND #19 (240)

3. **Medline Search Strategy**

("acquired brain injury" OR "stroke" OR "parkinson's") AND ("music" OR "instrument" OR "playing" OR “rhythm”)AND ("digital" OR "online" OR "AI" OR "artificial intelligence" OR "VR" OR "virtual reality" OR "MR" OR "mixed reality" OR "metaverse")

1. acquired brain injury (3,198)
2. stroke (469,187)
3. parkinson's (172,672)
4. #1 OR #2 OR #3 (647,691)
5. music (36,519)
6. instrument (314,612)
7. playing (65,245)
8. rhythm (194,932)
9. #5 OR #6 OR #7 OR #8 (440,880)
10. digital (261,862)
11. online (323,894)
12. AI (234,260)
13. artificial intelligence (115,526)
14. VR (55,402)
15. virtual reality (24,947)
16. MR (551,660)
17. mixed reality (2,898)
18. metaverse (734)
19. #10 OR #11 OR #12 OR #13 OR #14 OR #15 OR #16 OR #17 OR #18 (1,447,194)
20. #4 AND #9 AND #19 (992)

4. **Google Scholar Search Strategy**

("acquired brain injury" OR "stroke" OR "parkinson's") AND ("music" OR "instrument" OR "playing" OR “rhythm”)AND ("digital" OR "online" OR "AI" OR "artificial intelligence" OR "VR" OR "virtual reality" OR "MR" OR "mixed reality" OR "metaverse")

1. acquired brain injury (5,470)
2. stroke (498,000)
3. parkinson's (226,000)
4. #1 OR #2 OR #3 (5,490)
5. music (515,000)
6. instrument (200,000)
7. playing (115,000)
8. rhythm (112,000)
9. #5 OR #6 OR #7 OR #8 (643,000)
10. digital (1,090,000)
11. online (707,000)
12. AI (276,000)
13. artificial intelligence (227,000)
14. VR (92,800)
15. virtual reality (156,000)
16. MR (361,000)
17. mixed reality (11,700)
18. metaverse (29,800)
19. #10 OR #11 OR #12 OR #13 OR #14 OR #15 OR #16 OR #17 OR #18 (4,240)
20. #4 AND #9 AND #19 (37)

5. **Web of Science Core Collection (SCIE, SSCI, AHCI, CPCI) Search Strategy**

("acquired brain injury" OR "stroke" OR "parkinson's") AND ("music" OR "instrument" OR "playing" OR “rhythm”)AND ("digital" OR "online" OR "AI" OR "artificial intelligence" OR "VR" OR "virtual reality" OR "MR" OR "mixed reality" OR "metaverse")

1. acquired brain injury (5,244)
2. stroke (295,341)
3. parkinson's (101,824)
4. #1 OR #2 OR #3 (384,197)
5. music (77,882)
6. instrument (492,927)
7. playing (2,252,859)
8. rhythm (92,006)
9. #5 OR #6 OR #7 OR #8 (2,872,667)
10. digital (667,736)
11. online (613,656)
12. AI (142,854)
13. artificial intelligence (140,107)
14. VR (39,807)
15. virtual reality (64,634)
16. MR (151,592)
17. mixed reality (10,803)
18. metaverse (3,542)
19. #10 OR #11 OR #12 OR #13 OR #14 OR #15 OR #16 OR #17 OR #18 (1,666,989)
20. #4 AND #9 AND #19 (335)
